# Early Detection of PRRSV Outbreaks in Breeding Herds by Monitoring Productivity and Electronic Sow Feed Data Using Univariate and Multivariate Statistical Process Control Methods

**DOI:** 10.1155/2024/9984148

**Published:** 2024-07-18

**Authors:** Mafalda Pedro Mil-Homens, Swaminathan Jayaraman, Kinath Rupasinghe, Chong Wang, Giovani Trevisan, Fernanda Dórea, Daniel C. L. Linhares, Derald Holtkamp, Gustavo S. Silva

**Affiliations:** ^1^ Department of Veterinary Diagnostic and Production Animal Medicine College of Veterinary Medicine Iowa State University, Ames, IA, USA; ^2^ Department of Statistics College of Liberal Arts and Sciences Iowa State University, Ames, IA, USA; ^3^ Swedish Veterinary Agency, Uppsala, Sweden; ^4^ Food and Agriculture Organization of the United Nations FAO, Rome, Italy

## Abstract

Porcine reproductive and respiratory syndrome virus (PRRSV) is one of the most impactful viruses in swine production worldwide. Early detection of PRRSV outbreaks is the first step in rapid disease response. A syndromic surveillance system can be implemented to detect early signs of PRRSV activity in breeding herds. This study aimed to integrate multiple data sources and test univariate and multivariate statistical process control charts to identify early indicators associated with PRRSV outbreaks in sow farms. From March 2022 until May 2023, 16 breed-to-wean swine farms were enrolled in the study. The following key clinical and productivity indicators associated with PRRSV outbreaks were investigated: number of abortions, number of dead sows, number of off-feed events, preweaning mortality rate (PWM), and percentage of neonatal losses. The PRRSV status for the herd was determined by reverse transcriptase polymerase chain reaction testing using processing fluid samples, and it was considered as the reference to calculate the performance of the surveillance system. The exponentially weighted moving average (EWMA), cumulative sum (CUSUM), multivariate exponentially weighted moving average (MEWMA), and multivariate cumulative sum (MCUSUM) were the methods used to detect significant changes in the aforementioned parameters following the PRRSV outbreaks. Using the EWMA model, the indicators with the highest early detection rates were PWM followed by the abortions (71% and 64%, respectively), with the models raising alarms 4 weeks earlier on average than the processing fluids, respectively. For the CUSUM model, the weekly number of PWM, followed by abortions, were the indicators with the highest early detection rates (71% and 64%, respectively), with the models raising alarms 4 weeks earlier on average than the processing fluids for both indicators. Concerning the multivariate models, the MEWMA model with higher early detection used the PWM and neonatal losses (86%), with the models raising alarms 4 weeks earlier on average than the processing fluids, with the models raising alarms 3.5 weeks earlier on average than the processing fluids. For the MCUSUM, the model with higher early detection used PWM and neonatal losses (86%), with the models raising alarms 4.3 weeks earlier on average than the processing fluids. The models with the earliest time to detect signs associated with a PRRSV outbreak and with the lowest false negative and false positive were the multivariate models, MEWMA and the MCUSUM, using the combination of PWM and neonatal losses. Thus, monitoring multiple indicators outperformed the univariate models. With that, using multivariate models is the best option for disease surveillance using indicators, and it allows the decision-makers to investigate potential outbreaks earlier.

## 1. Introduction

Porcine reproductive and respiratory syndrome virus (PRRSV) has had a big impact on swine production worldwide [1, 2, 3], with annual losses above $500 million in the United States [4, 5]. The use of diagnostic methods to implement PRRSV surveillance systems at farms has been changing and evolving over the years. Several works have been published to test and compare different sample types, such as oral fluids [6, 7], family oral fluids [8], processing fluids [9], or tongue tips [10]. In the realm of sow farm management, a discernible shift is observed as operations transition from continuous diagnostic surveillance to a more strategic reliance on key production indicators to detect signs potentially associated with disease outbreaks. This becomes particularly pertinent once a sow farm achieves stability, or according to the American Association of Swine Veterinarians (AASV) classification, when the farm is classified as status II, i.e., consistently producing PRRSV-negative pigs at weaning [11]. Syndromic surveillance, characterized by detecting clinical case features before confirmed diagnoses, is central to this paradigm change [12]. Weekly production indicators serve as the linchpin for this refined approach, systematically monitoring sow health, reproduction, and other critical factors. The systematic surveillance of these indicators facilitates the early detection of deviations from normal productivity, acting as triggers for in-depth investigations and allowing for a more proactive response to potential challenges. This transition not only reduces the need for constant, resource-intensive diagnostics but also ensures that emerging issues are identified and addressed promptly, contributing to a more efficient and targeted sow farm management strategy. Control charts have been an object of interest in production systems to monitor deviations in data. This has been mentioned in literature for years, although it is not routinely used at the farms [13]. These charts have been tested in swine production [14, 15, 16], dairy production [17, 18], and cattle production [19] to monitor the patterns of production and potential signs of the introduction of pathogens. The following studies showed that the use of statistical process control (SPC) charts was useful in detecting significant deviations in subclinical and clinical disease and in productivity indicators. Li et al. [20] used SPC charts to monitor deviations in feeding levels during the dry period in cows to determine if the cow was at risk of having postpartum ketosis, and despite not having good results for the diagnosis of disease, it helped in the development of a program for nutrition guidance. In another study, it was observed that using the cumulative sum chart (CUSUM) to detect deviations in water intake in calves was successful in detecting sick calves with an average of 3.1 ± 8.8 days before the farm personnel could detect signals of disease [21].

Concerning swine and PRRSV surveillance, a study showed that by using univariate exponentially weighted moving averages (EWMA) to monitor the number of abortions, deviations associated with PRRSV outbreak could be detected 4 weeks compared to the diagnostic tests [15]. Another study used univariate SPC charts and simulated PRRSV outbreaks, demonstrated that the system provided early detection of unexpected deviations [16]. Both studies used sow production data, but pathogens can affect other indicators, such as feed consumption and water intake, since animals have a variety of clinical signs when facing disease challenges [21, 22]. These studies suggest the need to integrate different data sources to assess patterns of multiple production indicators, feed consumption, and water intake indicators to understand the impact of disease on the animal's performance and to identify deviations from what is expected, facilitating the early detection of disease outbreaks.

Monitoring productivity indicators in the swine industry is mostly done by assessing production indicators individually [15, 16, 23, 24]. These univariate models have some disadvantages because they are not capable of aggregating information from multiple variables, the model is restricted to monitoring individual variables, and they also have a limited capability in detecting deviations when variables are correlated. A way of overcoming these issues is by using multivariate control charts. These methods allow for a more comprehensive assessment of the behavior process. They may also be more effective at detecting deviations, because they consider the structure of correlations between variables via the covariance matrix [25]. Concerning the use of these models in animal production, a few studies have already been reported demonstrating the usefulness of monitoring bovine for early detection of illness [18, 26]. With that, there exists a significant opportunity within the swine production industry to enhance health monitoring and production standards by employing productivity indicators.

This study aimed to test univariate and multivariate SPC charts to early identify deviations in production indicators associated with PRRSV outbreaks in sow farms.

## 2. Materials and Methods

### 2.1. Study Design and Eligibility Criteria

This study was conducted as a prospective observational study where swine breeding herds were enrolled and monitored over time. From March 2022 until May 2023, a convenience sample of 16 swine breeding herds located in the United States Midwest (study population) were enrolled in the study. Production systems willing to share the data required to assess herd-level sensitivity of different surveillance methods were invited. The eligibility criteria were (a) willing to share the weekly sow farm production performance report, (b) being negative for PRRSV by reverse transcriptase polymerase chain reaction (RT-qPCR) using processing fluids, (c) sow farms weaning negative pigs (with farm status II, III, or IV according to the AASV guidelines), (d) willing to perform or share weekly diagnostic results using processing fluids, and (e) using individual electronic sow feeding (ESF) throughout the gestation phase.

### 2.2. Productivity and Feed Data

Feed data was sent daily or weekly, and productivity indicators data was sent weekly. The sow productivity indicators were obtained from the keeping recording systems used by the study participants (PigKnows^©^ and Maximus^©^). The electronic feed data was obtained from the farms using the Gestal^©^ or Maximus^©^ ESF systems. The sow productivity indicators evaluated were preweaning mortality rate, number of abortions, number of dead sows, and percentage of neonatal losses. These indicators were chosen according to the clinical signs most related to a PRRSV outbreak [27]. Concerning the feed data, the indicators used were the weekly number of off-feed events (the number of times the feeding machine did not read feed consumption in 24 hr for a specific sow) and the weekly feed intake [28]. A variable called “difference from the previous week” (DFPW) was created for each indicator. For this variable, the difference between the value of an indicator from 1 week to the previous week was determined (Table 1). This variable was created to test if the change between weeks in the productivity and feed indicators could be useful for disease surveillance.

### 2.3. Farm Status and Definition of an Outbreak

At the beginning of the study, all farms were classified as status II, III, or IV based on the AASV guidelines for PRRSV classification [11]. All farms enrolled submitted their processing fluids every week to the Iowa State University Veterinary Diagnostic Laboratory (VDL). At the VDL, the samples were aggregated in a weekly pool and tested by RT-qPCR. An outbreak was defined if the farm had a PRRSV RT-qPCR positive result.

### 2.4. Statistical Analysis

For this study, 14 herd-outbreaks were identified according to the outbreak definition. The unit of analysis was the herd week. If a productivity or feed indicator did not have enough data to make the 21 weeks of baseline or if the data had alarms during the baseline, that indicator was not used for the analysis. Four different SPC methods were tested in this study. The EWMA, the CUSUM, the multivariate exponentially weighted moving average (MEWMA), and the multivariate cumulative sum (MCUSUM). These four models were chosen for this study because they all are used in time series analysis and anomaly detection. All statistical analyses were implemented in the R statistical programming environment, R Core Team (2021) version 4.1.1 (2021-08-10).

### 2.5. Univariate Models

EWMA: The EWMA is a statistical method used for time-series data analysis that uses a smoothing parameter that provides the weight to the average. Two different smoothing parameters, 0.4 and 0.8, and standard deviations, 3.0 and 3.5, were used based on the literature [15, 16, 29]. The baseline was built within 21 weeks without any alarms before the outbreak. The package *qcc* (version 2.7, Scrucca, L. 2004) from R was used to implement the model.

CUSUM: The CUSUM is a statistical method used to monitor the cumulative deviation of a target value over time [29]. The model was built with a decision interval of 5 and 8 (number of standard errors of the summary statistics at which the CUSUM is out of control) and a shift detection of 1 and 0.5 (measured in standard errors of the summary statistics) for the CUSUM as default in the package *qcc* (version 2.7, Scrucca, L. 2004) from R. The baseline was built within 21 weeks without any alarms before the outbreak.

### 2.6. Multivariate Models

MEWMA: The model was built using the mult.chart() function from package *MSQC* (version 1.1.0, Santos-Fernandez 2022) from R. A smoothing parameter of *λ* = 0.4 and *λ* = 0.8 [26] and a baseline of 21 weeks were used.

MCUSUM: The model was built using the mult.chart() function from package *MSQC* (version 1.1.0, Santos-Fernandez 2022) from R. A target value of *h* = 3.5 and *h* = 5.5, an *α* = 0.05, and control limits of *k* = 0.5 [30] were used with a baseline of 21 weeks.

For each multivariate model (MEWMA and MCUSUM), the following combination of productivity and feed indicators were tested: (a) the number of abortions and number of dead sows, (b) the number of abortions and the number of off-feed events, (c) the number of abortions and preweaning mortality rate (PWM), (d) the number of abortions and neonatal losses, (e) the number of dead sows and the number of off-feed events, (f) the number of dead sows and PWM, (g) the number of dead sows and neonatal losses, (h) the number of off-feed events and PWM, (i) the number of off-feed events and neonatal losses, (j) the PWM and the neonatal losses, and (k) the same combinations for the DFPW variables were used for the analysis.

### 2.7. Outcomes

The outcomes were the percentage of early detections and time to detect PRRSV outbreaks for each productivity and feed indicator. Early detection was defined as identifying an outbreak before changing the PRRSV status based on processing fluids results by RT-qPCR testing. Control charts are categorized based on their average run length (ARL). The ARL of a chart represents the anticipated number of samples to be collected before the chart signals the occurrence of a shift in the process [31]. In the context of this study, which was defined as the time to detect disease, the interval in weeks between the week the model raised an alarm and the week of the change in PRRSV status based on processing fluids.

### 2.8. Model Performance

The performance was calculated by comparing the models' alarms (deviations of the indicators from the control limits) with the results from the RT-qPCR testing using processing fluids. Each farm was classified as PRRSV positive or negative based on the results from the diagnostics, and each week had a signal of 1 (significant deviation) or 0 (no deviation) according to the results from the models. Then, overall relative sensitivity, relative specificity, and accuracy were calculated using 2 × 2 tables in Excel. If a model did not raise the alarm in the week, and the farm had PRRSV positive results using the RT-qPCR testing in the same week or the previous 8 weeks the farm had PRRSV positive results using the RT-qPCR testing, that was considered a false negative. If the model raised alarms and the farm had no PRRSV positive results using the RT-qPCR testing, that was considered a false positive.

## 3. Results

Sixteen farms with a range of breeding inventory between 2,215 and 11,055 sows (median of 4,990 sows) were enrolled in the project. Twelve farms had at least one outbreak between 2020 and 2023. The other four did not have any PRRSV outbreaks. A total of 14 outbreaks were reported during this period. Table S1 in the supplementary material describes the number of weeks monitored per productivity indicator for EWMA and CUSUM, and Table S2 describes the number of weeks monitored per productivity indicator for the MEWMA and MCUSUM.

### 3.1. EWMA

The weekly abortions, followed by the PWM, were the indicators with the highest detection rates, 71% and 86%, respectively. PWM was the variable with the highest early detection rate (71%). In at least one outbreak, the EWMA model detected signs associated with PRRSV outbreaks 8 weeks before the sow farm reported a change in PRRSV status using processing fluids for all indicators. The smoothing parameter and sigma combination with the lowest false negatives were *λ* = 0.4 and *σ* = 3.  Table 2 summarizes the early detection and model performance for the number of abortions, the number of dead sows, PWM%, neonatal losses%, and off-feed events. Considering the DFPW variables, the DFPW of off-feed events was the indicator with the best performance, accuracy (96%), relative sensitivity (56%), and relative specificity (100%). Table S3 in the supplementary material summarizes the early detection and model performance for the DFPW variables.

### 3.2. CUSUM

The weekly number of PWM, followed by abortions, were the indicators with the highest early detection rates, 71% and 64%, respectively. Using the number of abortions, PWM, and dead sows, the CUSUM model detected signs associated with PRRSV outbreaks 8 weeks before the sow farm reported a change in PRRSV status using processing fluids in at least one outbreak. The decision interval of 5 and the shift detection of 1 performed better, with a higher detection rate and lower false negative rates than the decision interval of 8 and the shift detection of 0.5.  Table 2 summarizes the early detection and model performance for each productivity indicator using the CUSUM. Considering the DFPW variables, the DFPW of neonatal losses was the indicator with the best performance, accuracy (97%), relative sensitivity (50%), and relative specificity (98%). For the variables representing the DFPW, Table S4 in the supplementary material summarizes the results.

### 3.3. MEWMA

The model using weekly PWM and neonatal losses had the highest early detection rate (86%), followed by the models using off-feed events and dead sows or off-feed events and neonatal losses (both 78%). Using the smoothing parameter of 0.4 performed better, with a higher detection rate and lower false negative rates, than the smoothing parameter of 0.4.  Table 3 summarizes the early detection and model performance for each production indicator. Considering the DFPW variables, the DFPW of off-feed and abortions was the indicator with the best performance, accuracy (96%), relative sensitivity (63%), and relative specificity (97%). Table S5 in the supplementary material summarizes the results from the DFPW variables.

### 3.4. MCUSUM

The models using weekly PWM and neonatal losses, and abortions and dead sows, and abortions and neonatal losses had the highest early detection rate (86%), followed by the model using dead sows and neonatal losses with an early detection rate of 79%. A target value of *h* = 3.5, *α* = 0.05, and *k* = 0.5 performed better, with a higher detection rate and lower false negative rates than the combination of parameters of *h* = 5.5, *α* = 0.05, and *k* = 0.5.  Table 3 summarizes the early detection and model performance for each production indicator. Considering the DFPW variables, the DFPW of abortions and neonatal losses was the indicator with the best performance, accuracy (92%), relative sensitivity (92%), and relative specificity (92%). Table S6 in the supplementary material summarizes the results from the DFPW variables.

Based on the relative sensitivity and relative specificity of detection, the best models were MCUSUM and MEWMA using PWM and neonatal losses, with the model detecting 13 out of 14 outbreaks, with sensitivity 93% and specificity 86% for the MCUSUM and sensitivity 93% and specificity 88% for the MEWMA.

A comparison of multivariate models with high performance to multiple univariate models was done. The comparison included a variety of combinations, such as univariate and univariate and multivariate models (e.g., EWMA PWM and EWMA neonatal losses and MEWMA with PWM and neonatal losses), with the results in Table S7 in the supplementary material, and univariate or univariate and multivariate models (e.g., EWMA PWM or EWMA neonatal losses and MEWMA with PWM and neonatal losses), with the results on Table S8 in the supplementary material, to determine whether the both univariate models would have an alarm when the multivariate model also had an alarm or if only one of the univariate models had an alarm when the multivariate model had an alarm.

## 4. Discussion

The results indicated that integrating multiple data sources using productivity and feed indicators can detect early signs of PRRSV outbreaks, and it is helpful to understand the impact that a disease outbreak has on productivity. Relying solely on diagnostics using processing fluids may not enable producers to detect signs early, as observed in studies where signs appeared around 4 weeks before diagnosis using SPC charts [15, 31], similar to the findings of this study, where most indicators had an average of early detection of 4 weeks. Additionally, implementing SPC models using productivity indicators, as supported by these studies, offers valuable information for managing clinical cases, subclinical cases, and deviations in productivity.

The EWMA allows to highlight of short-term trends and identify changes in data patterns, being of great use when the impact of older data points needs to be reduced [29]. CUSUM is helpful in the identification of gradual or abrupt changes in the data and is useful when the deviations in the process are not easily detectable [29, 32]. Krieter et al. [33] applied EWMA and CUSUM charts to develop a method that detects emerging process changes in swine production using simulated data; both charts had similar performance and showed to be useful to trace changes in production; another study showed that using a combination of a linear model and a CUSUM chart was useful in monitoring the condition of young pigs by measuring water intake in real-time, with the model signaling sick pigs 1 day before the clinical signs [34]. For this study, both CUSUM and the EWMA showed that using univariate SPC charts can be helpful in monitoring data to help decision-makers in the field to detect early signs of PRRSV outbreaks, with abortions and PWM being the best indicators (highest detection, lowest false negatives and false positives). However, when a PRRSV outbreak occurs, multiple indicators can be affected, and the model performance might be affected when we look at more than one variable at the same time.

Using multivariate models that incorporate different sow productivity indicators and off-feed events demonstrated that looking at more than one indicator is useful in detecting early signs of PRRSV introduction. MEWMA helps identify correlations and comovements between indicators [35]. Concerning MCUSUM, this is helpful when we analyze data with interconnected indicators, and the purpose is to collectively identify shifts in the mean of those indicators [30]. The use of MEWMA and MCUSUM in swine production to detect alarms associated with PRRSV outbreaks has not yet been explored. A study using simulated epidemics in cattle showed that using the MEWMA model always detected the epidemics earlier than the MCUSUM [26]. In this study, the average early detection was around 4 weeks for both MEWMA and MCUSUM, with the combination of PWM and neonatal losses being the best combination of indicators in both models, with an early detection rate of 93%. This combination of variables was the best in the multivariate models; one reason might be because the disease affects the most the animals that are in the womb in the late stages of gestation, leading to stillborn and mummies, and it also has big effects on animals that are at an early stage in life (from birth to weaning) [36, 37]. Comparing the univariate to the multivariate models, this study findings were similar to the results of a study that compared univariate and multivariate models and concluded that the multivariate models had increased prediction capability, being able to better track significant changes between the observed process and the expected process behaviors [38].

This study calculated model performance (relative sensitivity, relative specificity, accuracy) using the swine industry's commonly used diagnostic sample for PRRSV monitoring, the processing fluids [11]. False positives can occur due to external factors not related to a disease, such as vaccination, problems in the electronic sow feeder, moving sows, and weather events, among others. When these false positives occur, it is easier to associate one of these events to the alarm raised by the model, or the sows can be tested just to be sure that the result is actually negative. On the other hand, when we have false negatives, and the sows are not yet showing clinical signs, then the diagnostic tests will be run later than they should be. With that, it was observed that the models used for this study had low false negatives when compared to the false positives, except for the DFPW variables. The false positives were the highest in the univariate CUSUM model, and that may be because the CUSUM model is more sensitive to small shifts because it updates the CUSUMs of deviations of the individual data points from the target mean [32]. Testing different combinations of the parameters of decision intervals and shift detection can be helpful in fine-tuning the model, as it was observed by Krieter et al. [33].

Concerning data reliability, the data utilized in this study was drawn from commonly employed sources within the swine industry. The reliance on weekly data instead of daily data may introduce potential gaps in capturing nuanced fluctuations, possibly obscuring short-term trends or anomalies. Exploring the feasibility of incorporating daily data collection may provide a more detailed understanding of sow behavior and health status. Moreover, the susceptibility to human error during data collection could introduce inaccuracies. Despite these challenges, it's essential to underscore that the data utilized in this study remains the most comprehensive and widely adopted representation of swine production dynamics within the industry. By leveraging established recording systems and electronic feed data sources such as PigKnows^©^, Maximus^©^, Gestal^©^, and others, the dataset reflects the realities of on-farm operations. Consequently, field veterinarians and producers rely on these datasets to detect anomalies, track trends, and inform decision-making processes to optimize herd health and performance.

A limitation of this study was the assumption that the gold standard for PRRSV diagnostics in sow farms was testing processing fluids. This type of sample is commonly used to classify the PRRSV status of farms, but it targets piglets and does not include the population of breeding females. Considering the specificity and sensitivity of the models, these were high, and that can be due to the high number of negative weeks being monitored and the low number of PRRSV outbreaks (*n* = 14) during the investigation period. Another limitation of this study was that the diagnostic data was pooled by week, and there were no records from the farm of the number of rooms used for the sampling; there was only the information on the pooled results of the RT-qPCR.

We have shown that it is feasible to use multivariate SPC methods in surveillance systems with productivity indicators. For future research, it would be valuable to examine data at different levels of detail (daily and weekly) in the breeding herd, to test different machine learning techniques to improve the precision of outbreak detection, and to apply the same approach of using productivity data to establish a surveillance system in the wean-to-finish phase.

## 5. Conclusion

In conclusion, the results showed that monitoring indicators from multiple data sources resulted in the early detection of PRRSV outbreaks, with multivariate models having the best model performance. With that, using multivariate models for surveillance of PRRSV outbreaks showed improvement compared to univariate models previously tested with the same purpose. Furthermore, looking at this type of data allows the decision-makers to investigate potential outbreaks earlier, providing them with valuable information to intervene, plan disease control and elimination, and to avoid spreading disease to other sites.

## Figures and Tables

**Table 1:** Description of the indicators used to calculate time to detect PRRSV-outbreak on the study herds.

Indicator	Description
Abortions	The number of abortions per week (*n*)
Dead sows	The number of dead sows per week (*n*)
Preweaning mortality (PMW)	The percentage of dead pigs from birth to weaning (%)
Neonatal losses	The difference between total born and born alive per week (%)
Off-feed events	The number of times the electronic sow feeder does not have a reading of feed consumption per week (*n*)

**Table 2:** Description of the results of detection and performance of the univariate models EWMA and CUSUM for abortions, dead sows, PWM, neonatal losses, and off-feed events, using different combinations of parameters.

Metric	Model	Abortions	PWM	Neonatal losses	Dead sows	Off-feed events
Detection	EWMA *σ* = 3.0 and *λ* = 0.4	86% (12/14)	71% (10/14)	50% (7/14)	50% (7/14)	33% (3/9)
	EWMA *σ* = 3.5 and *λ* = 0.4	86% (12/14)	57% (8/14)	64% (9/14)	36% (5/14)	44% (4/9)
	EWMA *σ* = 3.0 and *λ* = 0.8	50% (7/14)	50% (7/14)	57% (8/14)	36% (5/14)	11% (1/9)
	EWMA *σ* = 3.5 and *λ* = 0.8	71% (10/14)	57% (8/14)	57% (8/14)	43% (6/14)	44% (4/9)
	CUSUM decision interval 5 and shift 1	79% (11/14)	71% (10/14)	43% (6/14)	50% (7/14)	22% (2/9)
	CUSUM decision interval 8 and shift 0.5	64% (9/14)	71% (10/14)	50% (7/14)	50% (7/14)	11% (1/9)

Early detection	EWMA *σ* = 3.0 and *λ* = 0.4	64% (9/14)	71% (10/14)	43% (6/14)	43% (6/14)	33% (3/9)
	EWMA *σ* = 3.5 and *λ* = 0.4	64% (9/14)	57% (8/14)	50% (7/14)	36% (5/14)	44% (4/9)
	EWMA *σ* = 3.0 and *λ* = 0.8	36% (5/14)	43% (6/14)	43% (6/14)	36% (5/14)	11% (1/9)
	EWMA *σ* = 3.5 and *λ* = 0.8	50% (7/14)	50% (7/14)	36% (5/14)	36% (5/14)	44% (4/9)
	CUSUM decision interval 5 and shift 1	64% (10/14)	71% (10/14)	43% (6/14)	50% (7/14)	22% (2/9)
	CUSUM decision interval 8 and shift 0.5	64% (9/14)	71% (10/14)	50% (7/14)	50% (7/14)	11% (1/9)

Average of early signs	EWMA *σ* = 3.0 and *λ* = 0.4	−3.7	−4.3	−4.5	−4.1	−4.7
	EWMA *σ* = 3.5 and *λ* = 0.4	−3.6	−4.2	−4.7	−4.2	−4.5
	EWMA *σ* = 3.0 and *λ* = 0.8	−1.8	−3.9	−4.8	−3.8	−4.5
	EWMA *σ* = 3.5 and *λ* = 0.8	−2.7	−4.3	−4.7	−4.5	−4.5
	CUSUM decision interval 5 and shift 1	−4.4	−4.3	−4.5	−4.2	−4.3
	CUSUM decision interval 8 and shift 0.5	−4.3	−4.4	−4.3	−4.4	−4.5

Range early signs ^*∗*^	EWMA *σ* = 3.0 and *λ* = 0.4	−8 to −1	−8 to −1	−8 to −1	−8 to −1	−8 to −1
	EWMA *σ* = 3.5 and *λ* = 0.4	−8 to −1	−8 to −1	−8 to −1	−8 to −1	−8 to −1
	EWMA *σ* = 3.0 and *λ* = 0.8	−4 to −1	−8 to −1	−8 to −1	−8 to −1	−8 to −1
	EWMA *σ* = 3.5 and *λ* = 0.8	−8 to −1	−8 to −1	−8 to −1	−8 to −1	−8 to −1
	CUSUM decision interval 5 and shift 1	−8 to −1	−8 to −1	−8 to −1	−8 to −1	−8 to −1
	CUSUM decision interval 8 and shift 0.5	−8 to −1	−8 to −1	−8 to −1	−8 to −1	−8 to −1

FN ^*∗∗*^ %	EWMA *σ* = 3.0 and *λ* = 0.4	14.3	28.6	50.0	50.0	66.7
	EWMA *σ* = 3.5 and *λ* = 0.4	14.3	42.9	35.7	61.5	55.6
	EWMA *σ* = 3.0 and *λ* = 0.8	50.0	50.0	42.9	64.3	88.9
	EWMA *σ* = 3.5 and *λ* = 0.8	28.6	42.9	42.9	57.1	55.6
	CUSUM decision interval 5 and shift 1	21.4	28.6	57.1	50.0	77.8
	CUSUM decision interval 8 and shift 0.5	18.2	28.6	36.4	50.0	75.0

FP ^*∗∗*^ %	EWMA *σ* = 3.0 and *λ* = 0.4	5.7	6.5	17.6	14.1	20.0
	EWMA *σ* = 3.5 and *λ* = 0.4	4.9	11.6	17.7	12.3	7.4
	EWMA *σ* = 3.0 and *λ* = 0.8	1.7	10.0	16.4	7.5	11.8
	EWMA *σ* = 3.5 and *λ* = 0.8	3.3	8.5	10.2	6.5	0.0
	CUSUM decision interval 5 and shift 1	12.4	20.1	17.6	16.9	10.5
	CUSUM decision interval 8 and shift 0.5	15.6	23.2	23.9	22.2	8.8

Acc ^*∗∗*^ %	EWMA *σ* = 3.0 and *λ* = 0.4	87.3	92.6	81.6	85.2	76.3
	EWMA *σ* = 3.5 and *λ* = 0.4	94.1	87.7	82.0	86.9	89.7
	EWMA *σ* = 3.0 and *λ* = 0.8	97.2	89.2	83.0	91.4	76.7
	EWMA *σ* = 3.5 and *λ* = 0.8	96.3	90.8	89.1	92.6	96.6
	CUSUM decision interval 5 and shift 1	87.4	79.7	81.6	82.5	84.2
	CUSUM decision interval 8 and shift 0.5	84.4	76.7	75.9	77.1	89.1

Se ^*∗∗*^ %	EWMA *σ* = 3.0 and *λ* = 0.4	85.7	71.4	50.0	50.0	33.3
	EWMA *σ* = 3.5 and *λ* = 0.4	85.7	57.1	64.3	50.0	44.4
	EWMA *σ* = 3.0 and *λ* = 0.8	50.0	50.0	57.1	38.5	11.1
	EWMA *σ* = 3.5 and *λ* = 0.8	71.4	57.1	57.1	35.7	44.4
	CUSUM decision interval 5 and shift 1	78.6	71.4	42.9	50.0	22.2
	CUSUM decision interval 8 and shift 0.5	81.8	71.4	63.6	50.0	25.0

Sp ^*∗∗*^ %	EWMA *σ* = 3.0 and *λ* = 0.4	94.3	86.6	82.4	85.9	80.0
	EWMA *σ* = 3.5 and *λ* = 0.4	95.1	88.4	82.3	87.7	92.6
	EWMA *σ* = 3.0 and *λ* = 0.8	98.3	90.0	83.6	92.5	88.2
	EWMA *σ* = 3.5 and *λ* = 0.8	96.7	91.5	89.8	93.5	100.0
	CUSUM decision interval 5 and shift 1	87.6	79.9	82.4	83.1	89.5
	CUSUM decision interval 8 and shift 0.5	84.4	76.8	76.1	77.8	91.2

^*∗*^The range of early signs represents the time before the outbreak where the models raised alarms. For example, if at least one farm had an alarm 8 weeks before the positive diagnostic result, that would be considered as the minimum (−8 weeks);  ^*∗∗*^False negative rate = FN; False positive rate = FP; Accuracy % = Acc; Sensitivity % = Se; Specificity % = Sp.

**Table 3:** Description of the results of detection and performance of the multivariate models MEWMA and MCUSUM with different combinations of parameters.

Metric	Model	Abort and dead sows	Abort and off-feed	PWM and neonatal losses	Dead sows and off-feed	Dead sows and PWM	Dead sows and neonatal losses	Abort and PWM	Abort and neonatal losses	Off-feed and PWM	Off-feed and neonatal losses
Detection	MEWMA *λ* = 0.4	71% (10/14)	67% (6/9)	93% (13/14)	89% (8/9)	93% (13/14)	86% (12/14)	71% (10/14)	86% (12/14)	67% (6/9)	78% (7/9)
	MEWMA *λ* = 0.8	57% (8/14)	67% (6/9)	57% (8/14)	67% (6/9)	36% (5/14)	43% (6/14)	57.1% (8/14)	64% (9/14)	67% (6/9)	78% (7/9)
	MCUSUM *k* = 0.5 *h* = 3.5 *α* = 0.05	93% (13/14)	67% (6/9)	93% (13/14)	89% (8/9)	79% (11/14)	93% (13/14)	71% (10/14)	86% (12/14)	89% (8/9)	78% (7/9)
	MCUSUM *k* = 0.5 *h* = 5.5 *α* = 0.05	64% (9/14)	55.6% (5/9)	71.4% (10/14)	55.6% (5/9)	50% (7/14)	79% (11/14)	50% (7/14)	71% (10/14)	67% (6/9)	44% (4/9)

Early detection	MEWMA *λ* = 0.4	64% (9/14)	56% (5/9)	86% (12/14)	78% (7/9)	71% (10/14)	64% (9/14)	64% (9/14)	71% (10/14)	56% (5/9)	78% (7/9)
	MEWMA *λ* = 0.8	57% (8/14)	67% (6/9)	57% (8/14)	67% (6/9)	36% (5/14)	43% (6/14)	57.1% (8/14)	64% (9/14)	67% (6/9)	78% (7/9)
	MCUSUM *k* = 0.5 *h* = 3.5 *α* = 0.05	86% (12/14)	67% (6/9)	86% (12/14)	67% (6/9)	64% (9/14)	79% (11/14)	71% (10/14)	86% (12/14)	78% (7/9)	78% (7/9)
	MCUSUM *k* = 0.5 *h* = 5.5 *α* = 0.05	64% (9/14)	55.6% (5/9)	71.4% (10/14)	55.6% (5/14)	50% (7/14)	79% (11/14)	50% (7/14)	71% (10/14)	67% (6/9)	44% (4/9)

Average of early signs	MEWMA *λ* = 0.4	−3.8	−3.3	−4.3	−3.7	−4.4	−4.5	−4.0	−4.0	−3.8	−4.2
	MEWMA *λ* = 0.8	−3.4	−3.3	−3.7	−4.7	−4.3	−4.3	−3.4	−3.8	−4.1	−4.1
	MCUSUM *k* = 0.5 *h* = 3.5 *α* = 0.05	−4.4	−3.8	−4.4	−3.8	−4.0	−4.2	−4.1	−4.0	−3.7	−4.5
	MCUSUM *k* = 0.5 *h* = 5.5 *α* = 0.05	−4.4	−3.4	−4.3	−3.5	−4.1	−4.2	−4.1	−4.2	−3.7	−4.1

Range early signs ^*∗*^	MEWMA *λ* = 0.4	−8 to −1	−8 to −1	−8 to −1	−8 to −1	−8 to −1	−8 to −1	−8 to −1	−8 to −1	−8 to −1	−8 to −1
	MEWMA *λ* = 0.8	−8 to −1	−8 to −1	−8 to −1	−8 to −1	−8 to −1	−8 to −1	−8 to −1	−8 to −1	−8 to −1	−8 to −1
	MCUSUM *k* = 0.5 *h* = 3.5 *α* = 0.05	−8 to −1	−8 to −1	−8 to −1	−8 to −1	−8 to −1	−8 to −1	−8 to −1	−8 to −1	−8 to −1	−8 to −1
	MCUSUM *k* = 0.5 *h* = 5.5 *α* = 0.05	−8 to −1	−8 to −1	−8 to −1	−8 to −1	−8 to −1	−8 to −1	−8 to −1	−8 to −1	−8 to −1	−8 to −1

FN ^*∗∗*^ %	MEWMA *λ* = 0.4	23.1	33.3	7.1	11.1	7.1	14.3	28.6	14.3	14.3	22.2
	MEWMA *λ* = 0.8	33.3	33.3	33.3	25.0	50.0	40.0	38.5	18.2	25.0	22.2
	MCUSUM *k* = 0.5 *h* = 3.5 *α* = 0.05	7.1	33.3	7.1	11.1	15.4	7.1	28.6	28.6	11.1	22.2
	MCUSUM *k* = 0.5 *h* = 5.5 *α* = 0.05	30.8	44.4	16.7	28.6	46.2	8.3	41.7	16.7	25.0	42.9

FP ^*∗∗*^ %	MEWMA *λ* = 0.4	12.5	0.0	10.4	5.7	11.7	14.9	13.8	14.3	0.5	7.2
	MEWMA *λ* = 0.8	9.2	0.0	10.9	3.1	9.9	14.3	7.9	10.5	8.3	6.6
	MCUSUM *k* = 0.5 *h* = 3.5 *α* = 0.05	25.5	1.3	13.7	5.3	14.2	20.1	17.9	21.1	4.9	6.8
	MCUSUM *k* = 0.5 *h* = 5.5 *α* = 0.05	32.1	0.0	15.3	4.7	16.8	91.7	21.5	28.4	4.7	0.4

Acc ^*∗∗*^ %	MEWMA *λ* = 0.4	87.3	98.8	89.7	94.1	88.4	85.1	85.7	85.7	98.2	92.3
	MEWMA *λ* = 0.8	90.2	98.7	88.5	96.2	89.1	85.1	91.1	89.3	91.8	92.8
	MCUSUM *k* = 0.5 *h* = 3.5 *α* = 0.05	74.9	97.5	86.4	94.5	85.8	80.2	81.9	79.1	94.9	92.7
	MCUSUM *k* = 0.5 *h* = 5.5 *α* = 0.05	67.9	98.3	84.7	94.7	82.3	76.0	77.9	71.9	94.7	98.4

Se ^*∗∗*^ %	MEWMA *λ* = 0.4	76.9	67.7	92.9	88.9	92.9	85.7	71.4	85.7	85.7	77.8
	MEWMA *λ* = 0.8	66.7	66.7	66.7	75.0	50.0	60.0	61.5	81.8	75.0	77.8
	MCUSUM *k* = 0.5 *h* = 3.5 *α* = 0.05	92.9	66.7	92.9	88.9	84.6	92.9	71.4	85.7	88.9	77.8
	MCUSUM *k* = 0.5 *h* = 5.5 *α* = 0.05	69.2	55.6	83.3	71.4	53.8	91.7	58.3	83.3	75.0	57.1

Sp ^*∗∗*^ %	MEWMA *λ* = 0.4	87.5	100.0	89.6	94.3	88.3	85.1	86.2	85.7	91.2	92.3
	MEWMA *λ* = 0.8	90.8	100.0	89.1	96.9	90.1	85.7	92.1	89.5	91.7	93.4
	MCUSUM *k* = 0.5 *h* = 3.5 *α* = 0.05	74.5	98.7	86.3	94.7	85.8	79.9	82.1	78.9	95.1	93.2
	MCUSUM *k* = 0.5 *h* = 5.5 *α* = 0.05	67.9	100.0	84.7	95.3	83.2	75.6	78.5	71.6	95.3	99.6

^*∗*^The range of early signs represents the time before the outbreak where the models raised alarms. For example, if at least one farm had an alarm 8 weeks before the positive diagnostic result, that would be considered as the minimum (−8 weeks);  ^*∗∗*^False negative rate = FN; False positive rate = FP; Accuracy % = Acc; Sensitivity % = Se; Specificity % = Sp.

## Data Availability

The data used for this study was provided by different swine production systems and it was required of us to maintain the data unavailable for public view.

## Supplementary Materials

Supplementary Materials Supplementary material providing detailed tables (S1–S8) showcasing deviation detection, and performance metrics for both univariate (EWMA, CUSUM) and multivariate (MEWMA, MCUSUM) models.
