# Supplementary material for: Early Detection of PRRSV Outbreaks in Breeding Herds by Monitoring Productivity and Electronic Sow Feed Data Using Univariate and Multivariate Statistical Process Control Methods
Source: Transbound Emerg Dis. 2024 Jul 18;2024:9984148. doi: 10.1155/2024/9984148 (PMC12017042; doi:10.1155/2024/9984148)
Supplement: Supplementary Materials — Supplementary material providing detailed tables (S1–S8) showcasing deviation detection, and performance metrics for both univariate (EWMA, CUSUM) and multivariate (MEWMA, MCUSUM) models. [file 9984148.f1.docx]

# Supplementary material

**Table S1.** Table describing the number of weeks monitored per productivity indicator for EWMA and CUSUM

| **Productivity indicator** | **Model** | **Parameters** | **#Weeks monitored** |
| --- | --- | --- | --- |
| **Abortions** | **EWMA** | σ = 3.0 & λ = 0.4 | 835 |
|  |  | σ = 3.5 & λ = 0.4 | 835 |
|  |  | σ = 3.0 & λ = 0.8 | 621 |
|  |  | σ = 3.5 & λ = 0.8 | 835 |
|  | **CUSUM** | decision interval 5 and shift 1 | 885 |
|  |  | decision interval 8 and shift 0.5 | 885 |
| **Dead sows** | **EWMA** | σ = 3.0 & λ = 0.4 | 841 |
|  |  | σ = 3.5 & λ = 0.4 | 841 |
|  |  | σ = 3.0 & λ = 0.8 | 751 |
|  |  | σ = 3.5 & λ = 0.8 | 841 |
|  | **CUSUM** | decision interval 5 and shift 1 | 788 |
|  |  | decision interval 8 and shift 0.5 | 955 |
| **PWM** | **EWMA** | σ = 3.0 & λ = 0.4 | 741 |
|  |  | σ = 3.5 & λ = 0.4 | 741 |
|  |  | σ = 3.0 & λ = 0.8 | 717 |
|  |  | σ = 3.5 & λ = 0.8 | 785 |
|  | **CUSUM** | decision interval 5 and shift 1 | 808 |
|  |  | decision interval 8 and shift 0.5 | 808 |
| **Neonatal losses** | **EWMA** | σ = 3.0 & λ = 0.4 | 727 |
|  |  | σ = 3.5 & λ = 0.4 | 812 |
|  |  | σ = 3.0 & λ = 0.8 | 727 |
|  |  | σ = 3.5 & λ = 0.8 | 727 |
|  | **CUSUM** | decision interval 5 and shift 1 | 788 |
|  |  | decision interval 8 and shift 0.5 | 788 |
| **Off-feed** | **EWMA** | σ = 3.0 & λ = 0.4 | 131 |
|  |  | σ = 3.5 & λ = 0.4 | 162 |
|  |  | σ = 3.0 & λ = 0.8 | 75 |
|  |  | σ = 3.5 & λ = 0.8 | 162 |
|  | **CUSUM** | decision interval 5 and shift 1 | 138 |
|  |  | decision interval 8 and shift 0.5 | 138 |

**Table S2.** Table describing the number of weeks monitored per productivity indicator for MEWMA and MCUSUM

| **Productivity indicator** | **Model** | **Parameters** | **#Weeks monitored** |
| --- | --- | --- | --- |
| **Abort & Dead sows** | **MEWMA** | λ=0.4 | 805 |
|  |  | λ=0.8 | 805 |
|  | **MCUSUM** | k=0.5 h=3.5 α=0.05 | 803 |
|  |  | k=0.5 h=5.5 α=0.05 | 803 |
| **Abort & Off-feed** | **MEWMA** | λ=0.4 | 263 |
|  |  | λ=0.8 | 263 |
|  | **MCUSUM** | k=0.5 h=3.5 α=0.05 | 265 |
|  |  | k=0.5 h=5.5 α=0.05 | 265 |
| **PWM & Neonatal losses** | **MEWMA** | λ=0.4 | 754 |
|  |  | λ=0.8 | 754 |
|  | **MCUSUM** | k=0.5 h=3.5 α=0.05 | 739 |
|  |  | k=0.5 h=5.5 α=0.05 | 739 |
| **Dead sows & Off-feed** | **MEWMA** | λ=0.4 | 298 |
|  |  | λ=0.8 | 298 |
|  | **MCUSUM** | k=0.5 h=3.5 α=0.05 | 303 |
|  |  | k=0.5 h=5.5 α=0.05 | 303 |
| **Dead sows & PWM** | **MEWMA** | λ=0.4 | 648 |
|  |  | λ=0.8 | 648 |
|  | **MCUSUM** | k=0.5 h=3.5 α=0.05 | 639 |
|  |  | k=0.5 h=5.5 α=0.05 | 639 |
| **Dead sows & Neonatal losses** | **MEWMA** | λ=0.4 | 696 |
|  |  | λ=0.8 | 696 |
|  | **MCUSUM** | k=0.5 h=3.5 α=0.05 | 698 |
|  |  | k=0.5 h=5.5 α=0.05 | 698 |
| **Abort & PWM** | **MEWMA** | λ=0.4 | 591 |
|  |  | λ=0.8 | 591 |
|  | **MCUSUM** | k=0.5 h=3.5 α=0.05 | 668 |
|  |  | k=0.5 h=5.5 α=0.05 | 668 |
| **Abort & Neonatal losses** | **MEWMA** | λ=0.4 | 661 |
|  |  | λ=0.8 | 661 |
|  | **MCUSUM** | k=0.5 h=3.5 α=0.05 | 615 |
|  |  | k=0.5 h=5.5 α=0.05 | 615 |
| **Off-feed & PWM** | **MEWMA** | λ=0.4 | 138 |
|  |  | λ=0.8 | 138 |
|  | **MCUSUM** | k=0.5 h=3.5 α=0.05 | 303 |
|  |  | k=0.5 h=5.5 α=0.05 | 303 |
| **Off-feed & Neonatal losses** | **MEWMA** | λ=0.4 | 285 |
|  |  | λ=0.8 | 285 |
|  | **MCUSUM** | k=0.5 h=3.5 α=0.05 | 285 |
|  |  | k=0.5 h=5.5 α=0.05 | 285 |

**Table S3.** Table describing the results of detection and performance of the EWMA for the DFPW variables, using different combinations of sigmas and smoothing parameters.

| Productivity indicators | Sigma and smoothing parameter | # Weeks used | Detection | Early detection | Average of early signs | Range early sign * | FN**  % | FP**  % | Acc**  % | Se**  % | Sp**  % |
| --- | --- | --- | --- | --- | --- | --- | --- | --- | --- | --- | --- |
| DFPW of abort | σ = 3.0 & λ = 0.4 | 693 | 36% (5/14) | 29% (4/14) | -2 | -3 to -1 | 61.5 | 1.8 | 97.1 | 38.5 | 98.2 |
|  | σ = 3.5 & λ = 0.4 | 693 | 36% (5/14) | 29% (4/14) | -2.9 | -8 to -1 | 61.5 | 1.9 | 97.0 | 38.5 | 98.1 |
|  | σ = 3.0 & λ = 0.8 | 617 | 36% (5/14) | 29% (4/14) | -3 | -8 to -1 | 64.3 | 1.7 | 96.9 | 35.7 | 98.3 |
|  | σ = 3.5 & λ = 0.8 | 617 | 36% (5/14) | 29% (4/14) | -3 | -8 to -1 | 61.3 | 1.3 | 97.2 | 35.7 | 98.7 |
| DFPW of dead sows | σ = 3.0 & λ = 0.4 | 684 | 14% (2/14) | 7% (1/14) | -2 | -2 | 85.7 | 0.7 | 98.0 | 14.3 | 99.3 |
|  | σ = 3.5 & λ = 0.4 | 684 | 7% (1/14) | 0% (0/14) | 0 | 0 | 92.9 | 0.1 | 98.0 | 7.1 | 99.9 |
|  | σ = 3.0 & λ = 0.8 | 684 | 7% (1/14) | 0% (0/14) | 0 | 0 | 92.3 | 1.5 | 96.8 | 7.7 | 98.5 |
|  | σ = 3.5 & λ = 0.8 | 684 | 14% (2/14) | 7% (1/14) | -2 | -2 | 85.7 | 1.3 | 96.9 | 14.3 | 98.7 |
| DFPW of PWM | σ = 3.0 & λ = 0.4 | 714 | 14% (2/14) | 7% (1/14) | -1.5 | -2 to -1 | 85.7 | 1.1 | 97.2 | 14.3 | 98.9 |
|  | σ = 3.5 & λ = 0.4 | 714 | 14% (2/14) | 7% (1/14) | -1.5 | -2 to -1 | 85.7 | 1.4 | 96.9 | 14.3 | 98.6 |
|  | σ = 3.0 & λ = 0.8 | 714 | 29% (4/14) | 14% (2/14) | -1.7 | -2 to -1 | 69.2 | 0.9 | 97.9 | 30.8 | 99.1 |
|  | σ = 3.5 & λ = 0.8 | 714 | 7% (1/14) | 14% (2/14) | -1.5 | -2 to -1 | 85.7 | 1.1 | 97.2 | 14.3 | 98.9 |
| DFPW of Neonatal losses | σ = 3.0 & λ = 0.4 | 658 | 36% (5/14) | 21% (3/14) | -4.6 | -8 to -1 | 58.3 | 1.4 | 97.5 | 41.7 | 98.6 |
|  | σ = 3.5 & λ = 0.4 | 658 | 36% (5/14) | 21% (3/14) | -4.6 | -8 to -1 | 58.3 | 1.3 | 97.7 | 41.7 | 98.7 |
|  | σ = 3.0 & λ = 0.8 | 619 | 50% (7/14) | 21% (3/14) | -4.7 | -8 to -1 | 41.7 | 1.8 | 97.4 | 58.3 | 98.2 |
|  | σ = 3.5 & λ = 0.8 | 658 | 43% (6/14) | 14% (2/14) | -5.2 | -8 to -1 | 53.8 | 0.8 | 98.1 | 46.2 | 99.2 |
| DFPW of off-feed events | σ = 3.0 & λ = 0.4 | 247 | 56% (5/9) | 33% (3/9) | -3.3 | -8 to -1 | 44.4 | 0.0 | 98.3 | 55.6 | 100.0 |
|  | σ = 3.5 & λ = 0.4 | 248 | 44% (4/9) | 33% (3/9) | -3.6 | -8 to -1 | 55.6 | 0.4 | 97.9 | 44.4 | 100.0 |
|  | σ = 3.0 & λ = 0.8 | 247 | 44% (4/9) | 33% (3/9) | -3.4 | -8 to -1 | 55.6 | 0.4 | 97.5 | 44.4 | 99.6 |
|  | σ = 3.5 & λ = 0.8 | 248 | 56% (5/9) | 33% (3/9) | -3.7 | -7 to -1 | 44.4 | 0.4 | 97.9 | 55.6 | 99.6 |

* The range of early signs represents the time before the outbreak where the models raised alarms. For example, if at least one farm had an alarm 8 weeks before the positive diagnostic result, that would be considered as the minimum (-8 weeks).

** False Negative rate = FN; False Positive rate = FP; Accuracy % = Acc; Sensitivity % = Se; Specificity % = Sp.

**Table S4.** Table describing the results of detection and performance of the CUSUM for the DFPW variables.

| Productivity indicators | # Weeks used | Detection | Early detection | Average of early signs | Range early sign * | FN**  % | FP**  % | Acc**  % | Se**  % | Sp**  % |
| --- | --- | --- | --- | --- | --- | --- | --- | --- | --- | --- |
| DFPW of abort | 687 | 43% (6/14) | 29% (4/14) | -1.8 | -3 to -1 | 57.1 | 1.2 | 97.7 | 42.9 | 98.8 |
| DFPW of dead sows | 719 | 7% (1/14) | 7% (1/14) | -2 | -2 | 92.9 | 0.4 | 97.8 | 7.1 | 99.6 |
| DFPW of PWM | 755 | 29% (4/14) | 29% (4/14) | -5.4 | -8 to -2 | 71.4 | 2.5 | 96.2 | 28.6 | 97.5 |
| DFPW of Neonatal losses | 620 | 50% (7/14) | 21% (3/14) | -5.3 | -8 to -2 | 50.0 | 2.2 | 96.7 | 50.0 | 97.8 |
| DFPW of off-feed events | 273 | 11% (1/9) | 11% (1/9) | -7.5 | -8 to -7 | 88.9 | 2.3 | 94.7 | 11.1 | 97.7 |

* The range of early signs represents the time before the outbreak where the models raised alarms. For example, if at least one farm had an alarm 8 weeks before the positive diagnostic result, that would be considered as the minimum (-8 weeks).

** False Negative rate = FN; False Positive rate = FP; Accuracy % = Acc; Sensitivity % = Se; Specificity % = Sp.

**Table S5.** Table describing the results of detection and performance of the MEWMA for the DFPW variables.

| Productivity indicators | # Weeks used | Detection | Early detection | Average of early signs | Range early sign * | FN**  % | FP**  % | Acc**  % | Se**  % | Sp**  % |
| --- | --- | --- | --- | --- | --- | --- | --- | --- | --- | --- |
| DFPW of abort & DFPW dead sows | 443 | 43% (6/14) | 36% (4/14) | -1.9 | -3 to -1 | 57.1 | 3.8 | 94.5 | 42.9 | 96.2 |
| DFPW abort & DFPW off-feed | 268 | 56% (5/9) | 44% (4/9) | -3.5 | -8 to -1 | 37.5 | 3.2 | 95.7 | 62.5 | 96.8 |
| DFPW of PWM & DFPW Neonatal losses | 527 | 50% (7/14) | 29% (4/14) | -3.4 | -8 to -1 | 50.0 | 2.2 | 96.5 | 50.0 | 97.8 |
| DFPW of abort & DFPW PWM | 509 | 29% (4/14) | 29% (4/14) | -3.9 | -8 to -1 | 69.2 | 2.7 | 95.5 | 30.8 | 97.3 |
| DFPW of abort & DFPW Neonatal losses | 490 | 64% (9/14) | 50% (7/14) | -4.2 | -8 to -1 | 35.7 | 7.9 | 91.2 | 64.3 | 92.1 |
| DFPW of dead sows & DFPW PWM | 485 | 21% (3/14) | 21% (3/14) | -4.9 | -8 to -1 | 78.6 | 2.6 | 95.1 | 21.4 | 97.4 |
| DFPW of dead sows & DFPW Neonatal losses | 485 | 57% (8/14) | 43% (6/14) | -4.4 | -8 to -1 | 42.9 | 8.6 | 90.4 | 57.1 | 91.4 |
| DFPW of Off-feed & DFPW PWM | 297 | 44% (4/9) | 33% (3/9) | -3.3 | -7 to -1 | 55.6 | 3.6 | 94.8 | 44.4 | 96.4 |
| DFPW of Off-feed & DFPW Neonatal losses | 297 | 67% (6/9) | 44% (4/9) | -5 | -8 to -1 | 33.3 | 7.4 | 91.8 | 66.7 | 92.6 |
| DFPW of Off-feed & DFPW dead sows | 297 | 56% (5/9) | 44 % (4/9) | -6.25 | -8 to -3 | 44.4 | 3.7 | 94.9 | 55.6 | 96.3 |

* The range of early signs represents the time before the outbreak where the models raised alarms. For example, if at least one farm had an alarm 8 weeks before the positive diagnostic result, that would be considered as the minimum (-8 weeks).

** False Negative rate = FN; False Positive rate = FP; Accuracy % = Acc; Sensitivity % = Se; Specificity % = Sp.

.

**Table S6.** Table describing the results of detection and performance of the MCUSUM for the DFPW variables.

| Productivity indicators | # Weeks used | Detection | Early detection | Average of early signs | Range early sign * | FN**  % | FP**  % | Acc**  % | Se**  % | Sp**  % |
| --- | --- | --- | --- | --- | --- | --- | --- | --- | --- | --- |
| DFPW of abort & DFPW dead sows | 444 | 36% (5/14) | 29% (4/14) | -1.8 | -3 to -1 | 64.3 | 1.2 | 96.8 | 35.7 | 98.8 |
| DFPW abort & DFPW off-feed | 269 | 44% (4/9) | 33% (3/9) | -4.0 | -8 to -1 | 50.0 | 2.4 | 96.2 | 50.0 | 97.6 |
| DFPW of PWM & DFPW Neonatal losses | 528 | 50% (7/14) | 29% (4/14) | -3.4 | -8 to -1 | 50.0 | 1.6 | 97.1 | 50.0 | 98.4 |
| DFPW of abort & DFPW PWM | 509 | 50% (7/14) | 43% (6/14) | -3.9 | -8 to -1 | 50.0 | 2.5 | 96.1 | 50.0 | 97.5 |
| DFPW of abort & DFPW Neonatal losses | 491 | 86% (12/14) | 79% (11/14) | -4.2 | -8 to -1 | 7.7 | 8.3 | 91.8 | 92.3 | 91.7 |
| DFPW of dead sows & DFPW PWM | 486 | 36% (5/14) | 29% (4/14) | -4.8 | -8 to -1 | 64.3 | 2.6 | 95.5 | 35.7 | 97.4 |
| DFPW of dead sows & DFPW Neonatal losses | 486 | 71% (10/14) | 57% (8/14) | -4.4 | -8 to -1 | 28.6 | 8.8 | 90.6 | 71.4 | 91.2 |
| DFPW of Off-feed & DFPW PWM | 298 | 56% (5/9) | 44% (4/9) | -3.5 | -8 to -1 | 44.4 | 3.3 | 95.4 | 55.6 | 96.7 |
| DFPW of Off-feed & DFPW Neonatal losses | 298 | 78% (7/9) | 78% (7/9) | -3.8 | -8 to -1 | 22.2 | 8.0 | 91.5 | 77.8 | 92.0 |
| DFPW of Off-feed & DFPW dead sows | 298 | 33% (3/9) | 22% (2/9) | -3.4 | -7 to -1 | 66.7 | 2.1 | 95.9 | 33.3 | 97.9 |

* The range of early signs represents the time before the outbreak where the models raised alarms. For example, if at least one farm had an alarm 8 weeks before the positive diagnostic result, that would be considered as the minimum (-8 weeks).

** False Negative rate = FN; False Positive rate = FP; Accuracy % = Acc; Sensitivity % = Se; Specificity % = Sp.

**Table S7.** Table describing the results of detection and performance of multiple univariates and multivariate models. If the two univariate models had alarms and the multivariate model had an alarm, that was considered as an alarm.

| Models | Productivity indicators | Detection | Early detection | Average of early signs | Range early sign * | FN**  % | FP**  % | Acc**  % | Se**  % | Sp**  % |
| --- | --- | --- | --- | --- | --- | --- | --- | --- | --- | --- |
| EWMA & MEWMA | PWM & neonatal losses | 50% (7/14) | 36% (5/14) | -4.3 | -8 to -1 | 50.0 | 5.2 | 93.8 | 50.0 | 94.8 |
|  | Abortions & neonatal losses  **Abortions & neonatal losses** | 43% (6/14) | 29% (4/14) | -4.4 | -8 to -1 | 57.1 | 1.7 | 97.1 | 42.9 | 98.3 |
|  | Dead sows & neonatal losses  **Dead sows & neonatal losses** | 29% (6/14) | 14% (2/14) | -4.9 | -8 to -1 | 71.4 | 2.6 | 95.8 | 28.6 | 97.4 |
|  | Off-feed & dead sows  **Off-feed & dead sows** | 11% (1/9) | 11% (1/9) | -3.5 | -4 to -3 | 88.9 | 0.0 | 97.8 | 11.1 | 100.0 |
|  | Off-feed & neonatal losses  **Off-feed & neonatal losses** | 0% (0/9) | 0% (0/9) | 0 | 0 | 100.0 | 0.0 | 97.6 | 0.0 | 100.0 |
| CUSUM & MCUSUM | PWM & neonatal losses | 36% (5/14) | 36% (5/14) | -4.6 | -8 to -1 | 64.3 | 2.7 | 95.8 | 35.7 | 97.3 |
|  | Abort & neonatal losses  **Abort & neonatal losses** | 29% (4/14) | 21% (3/14) | -4.1 | -8 to -1 | 69.2 | 1.3 | 97.3 | 30.8 | 98.7 |
|  | Dead sows & neonatal losses  **Dead sows & neonatal losses** | 29% (4/14) | 29% (4/14) | -4.5 | -8 to -1 | 71.4 | 3.9 | 94.5 | 28.6 | 96.1 |
|  | Off-feed & dead sows  **Off-feed & dead sows** | 11% (1/9) | 11% (1/9) | -3.5 | -4 to -3 | 88.9 | 0.0 | 97.8 | 11.1 | 100.0 |
|  | Off-feed & neonatal losses  **Off-feed & neonatal losses** | 0% (0/9) | 0% (0/9) | 0 | 0 | 100.0 | 0.0 | 97.6 | 0.0 | 100.0 |

* The range of early signs represents the time before the outbreak where the models raised alarms. For example, if at least one farm had an alarm 8 weeks before the positive diagnostic result, that would be considered as the minimum (-8 weeks).

** False Negative rate = FN; False Positive rate = FP; Accuracy % = Acc; Sensitivity % = Se; Specificity % = Sp.

**Table S8.** Table describing the results of detection and performance of multiple univariates and multivariate models. If the one univariate model or the other univariate model and the multivariate model had an alarm, that was considered as an alarm.

| Models | Productivity indicators | Detection | Early detection | Average of early signs | Range early sign * | FN**  % | FP**  % | Acc**  % | Se**  % | Sp**  % |
| --- | --- | --- | --- | --- | --- | --- | --- | --- | --- | --- |
| EWMA  & MEWMA | PWM & neonatal losses | 79% (11/14) | 71% (10/14) | -4.2 | -8 to -1 | 21.4 | 6.8 | 92.8 | 78.6 | 93.2 |
|  | Abortions & neonatal losses | 71% (10/14) | 57% (8/14) | -3.9 | -8 to -1 | 23.1 | 4.8 | 94.9 | 76.9 | 95.2 |
|  | Dead sows & neonatal losses | 71% (10/14) | 42% (6/14) | -4.3 | -8 to -1 | 28.6 | 4.7 | 94.7 | 71.4 | 95.3 |
|  | Off-feed & dead sows | 56% (5/9) | 44% (4/9) | -3.9 | -7 to -1 | 44.4 | 0.0 | 99.1 | 55.6 | 100.0 |
|  | Off-feed & neonatal losses | 56% (5/9) | 44% (4/9) | -4.5 | -8 to -1 | 44.4 | 0.0 | 99.1 | 55.6 | 100.0 |
| CUSUM & MCUSUM | PWM & neonatal losses | 79% (11/14) | 71% (10/14) | -4.3 | -8 to -1 | 21.4 | 3.7 | 95.9 | 78.6 | 96.3 |
|  | Abort & neonatal losses | 71% (10/14) | 64% (9/14) | -3.9 | -8 to -1 | 28.6 | 1.3 | 98.1 | 71.4 | 98.7 |
|  | Dead sows & neonatal losses | 64% (9/14) | 64% (9/14) | -4.0 | -8 to -1 | 35.7 | 5.9 | 93.4 | 64.3 | 94.1 |
|  | Off-feed & dead sows | 33% (3/9) | 33% (3/9) | -3.8 | -7 to -1 | 66.7 | 0.0 | 98.7 | 33.3 | 100.0 |
|  | Off-feed & neonatal losses | 22% (2/9) | 22% (2/9) | -4.1 | -8 to -1 | 77.8 | 0.0 | 98.5 | 22.2 | 100.0 |

* The range of early signs represents the time before the outbreak where the models raised alarms. For example, if at least one farm had an alarm 8 weeks before the positive diagnostic result, that would be considered as the minimum (-8 weeks).

** False Negative rate = FN; False Positive rate = FP; Accuracy % = Acc; Sensitivity % = Se; Specificity % = Sp.
